# The Resuscitation, Equilibrium and De-escalation (RED) strategy: a phased, personalized hemodynamic support in children with sepsis

**DOI:** 10.3389/fped.2025.1530984

**Published:** 2025-01-29

**Authors:** Jaime Fernández-Sarmiento, Sushitra Ranjit, L. Nelson Sanchez-Pinto, Vinay M. Nadkarni, Roberto Jabornisky, Niranjan Kissoon

**Affiliations:** ^1^ Department of Critical Care Medicine and Pediatrics, Fundación Cardioinfantil-Instituto de Cardiología, Universidad de La Sabana, Bogotá, Colombia; ^2^ Department of Pediatric Intensive Care Unit, Apollo Children’s Hospital, Chennai, India; ^3^ Department of Pediatrics, Northwestern University Feinberg School of Medicine, Stanley Manne Childreńs Research Institute, Ann & Robert H. Lurie Children’s Hospital, Chicago, IL, United States; ^4^ Division of Critical Care Medicine, Department of Anesthesiology and Critical Care, The Children’s Hospital of Philadelphia, University of Pennsylvania Perelman School of Medicine, Philadelphia, PA, United States; ^5^ Department of Pediatrics, Facultad de Medicina, Universidad Nacional del Nordeste, Corrientes, Argentina; ^6^ Department of Pediatrics, Children's Hospital Research Institute, BC Children's Hospital, University of British Columbia, Vancouver, BC, Canada

**Keywords:** septic shock, children, guidelines, fluid bolus, adrenaline, mortality

## Abstract

Hemodynamic support in critically ill children with septic shock is a pervasive challenge in the intensive care settings. Cardiovascular involvement in sepsis entails both macro- and microcirculation abnormalities, with the main treatment objectives seeking to increase cardiac output and improve tissue perfusion, respectively. Fluid therapy and vasoactive drugs are cornerstone therapies for circulatory problems in sepsis. Fluid boluses are a common first-line treatment for actual and relative hypovolemia. However, their use has been linked to adverse events due to factors such as their composition, high volumes and rapid infusion rates, and the variable response of individual patients. Furthermore, they often have transient efficacy or lack of response in many patients. Vasoactive drugs are also often used late, which favors repetitive fluid boluses, leading to hypervolemia, tissue edema and worse outcomes. After the resuscitation phase, active fluid removal through diuresis or dialysis is increasingly being used in patients who receive fluid therapy, but it has not yet been standardized, and the safest and most effective strategies in children are still not known. We believe that these interventions for hemodynamic problems in sepsis offer an opportunity to personalize treatment and apply precision medicine strategies. Using a phased approach adapted to each patient's context and clinical condition can potentially improve outcomes. The proposed *Resuscitation*, *Equilibrium* and *De-escalation* (RED) strategy is a simplified phased hemodynamic management approach for patients with sepsis and septic shock. Our goal with the introduction of this concept is to organize and underscore the fact that the cardiovascular support of sepsis is dynamic and should be adapted to each individual and context.

## Introduction

Sepsis continues to be a public health problem with high morbidity and mortality, especially in countries with limited resources (1). Up to half of all sepsis-related deaths occur within the first 48 h, mainly due to refractory shock (2). The most recent pediatric sepsis management guidelines recommend considering the context and presence of hypotension when using fluid boluses as the first line of management for children with septic shock (3, 4). Today, the main research and development lines in children with sepsis-related hemodynamic abnormalities are aimed at evaluating fluid responsiveness indicators, hypervolemia associated with non-resuscitation fluids, early initiation of vasoactive agents, and fluid redistribution in children with sepsis (5–7).

We believe that the use of a structured, phased hemodynamic management approach could help improve outcomes in children with septic shock (8, 9). The approach to shock in adults was initially proposed in four phases, seeking to adapt the monitoring and treatment goal to each phase (Salvage, Optimization, Stabilization and De-escalation, known as SOSD) (10). This approach was later termed the resuscitation, optimization, stabilization and evacuation (ROSE) strategy, highlighting that hemodynamic resuscitation in shock is a *dynamic* concept (11). Streamlining and identifying each of these hemodynamic intervention stages in septic shock can provide clinicians with a more holistic approach and can help personalize treatments according to the clinical condition and timing of septic shock diagnosis (11).

However, while the *optimization* phase seeks to adjust hemodynamic support to improve perfusion, excessive reliance on macrocirculation parameters may not accurately reflect tissue perfusion. In addition, some macrocirculatory changes tend to occur late in pediatrics, as is the case of hypotension which, when present, indicates greater disease severity (12). The *stabilization* phase involves a continuous administration of fluids and vasopressors which may result in hypervolemia and pulmonary edema. Additional fluid boluses must be well justified and based on much more precise and specific monitoring. We believe that these two stages (*optimization* and *stabilization*) have common objectives aimed at seeking hemodynamic equilibrium in children with sepsis and could be simplified to a single phase. In pediatrics, the inclusion of both phases under the concept of “*equilibrium*” can facilitate continuous and adaptable clinical management, especially in critical care settings. Furthermore, it provides a simplified framework which may be useful for clinical practice, in which adherence and speed are essential. This approach is especially relevant for institutions with limited resources or less specialized staff, where simplified terminology can promote better outcomes.

Therefore, in this review, we propose a new pediatric strategy of Resuscitation, Equilibrium and De-escalation (RED) as an approach to circulatory shock which, adapted from ROSE, aims to be more personalized and updated with the most recent pathophysiological advances. The RED strategy seeks to make healthcare staff aware that the hemodynamic approach in sepsis must be dynamic rather than static. What may initially be helpful may be harmful in advanced stages of the disease. It also highlights the idea that the interventions should be structured and adapted to the patient's clinical condition and both macro- and micro-circulatory changes.

## The RED strategy

The Resuscitation, Equilibrium and De-escalation (RED) strategy involves a holistic, dynamic and updated approach to all the hemodynamic intervention phases in pediatric sepsis and septic shock. In addition to conventional management strategies that includes early recognition and initiation of antibiotics, a structured, phased approach allows the hemodynamic resuscitation phases or phenotypes in sepsis to be streamlined and personalized (Table 1).

**Table 1:** Objectives of the R.E.D. phases and monitoring strategies in septic shock.

Phase of R.E.D concept	Targets	Interventions	Monitoring strategies
1. Resuscitation	*Macrocirculation* - Optimize AP - Optimize CO *Microcirculation* - Optimize tissue perfusion	- Fluids bolus 10–20 ml/kg in patients with hypotension - Hypoperfusion with PICU: fluids bolus - Hypoperfusion without PICU: no fluids bolus - Inotropes - Vasopressors	- Clinical examination - Respiratory mechanics - Noninvasive or invasive arterial pressure/PP - Heart rate - CRT - Urine output - Lactate - Echocardiography - POCUS
2. Equilibrium	*Macrocirculation* - Provide organ support *Microcirculation* - Normalize tissue perfusion indices	- Fluids according to fluid responsiveness and tolerance. - Vasopressors - Inotropes - Avoid fluid creep - Monitor fluid balance	- Clinical examination - Respiratory mechanics - Arterial pressure/PP - Heart rate - Diastolic blood pressure (low in vasodilatory shock) - CRT - Lactate - Urine output - Advanced hemodynamic monitoring (minimally invasive CO) - ScvO_2_ and ΔP(v-a)CO_2_
3. De-escalation	*Macrocirculation* - Decreased organ support. *Microcirculation* - Limit exposure to high doses of fluids - Limit impact of accumulated fluids and tisular edema	- Monitor fluid balance - Fluid restriction in patients with fluid overload. - Decrease dose of vasopressors/inotropes or suspend - Fluid removal in case of tisular edema with positive fluid balance: diuretics, albumin, CRRT.	- Maintain existing monitoring - Clinical examination - Respiratory mechanics - Normal CRT prior to fluid removal. - Urine output - Lactate

AP, arterial pressure; CO, cardiac output; PICU, pediartric intensive care units; PP, pulse pressure; CRT, capillary refill time; POCUS, point-of-care ultrasonography; CRRT, continuos replacement renal therapy. ScvO_2_ central venous oxygen saturation. ΔP(v-a) CO_2_ central venous-to-arterial CO_2_ difference.

For this review and viewpoint, a systematic search of the PubMed, Embase and Cochrane Library databases was conducted up to July 2024. The search terms included “*pediatric sepsis*,” “*fluid therapy*,” “*vasopressors*”, “*shock management,*”, “*diuretics*”, “*hypervolemia*”, “*tolerance fluids*”, “*albumin*”, and “*fluid creep,*” combined using Boolean operators. Studies in English and Spanish that evaluated fluid and vasopressor management strategies in pediatric patients with sepsis were included. We included clinical trials, observational studies, systematic reviews, and opinion articles, while editorials, letters to the editor and case reports were excluded.

This RED strategy could help personalize interventions according to the patients' characteristics and clinical condition in all phases of circulatory failure in children with sepsis. Below, we present each of the proposed phases with an initial clinical case that illustrates the challenges and difficulties faced by clinicians in real-world practice.

**Figure 1:**
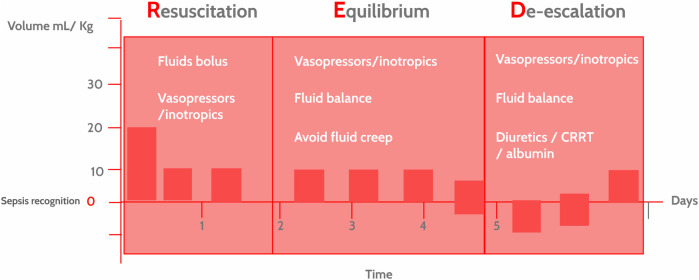
The resuscitation, equilibrium and De-escalation (RED) strategy in hemodynamic interventions in pediatric sepsis. Hemodynamic interventions in pediatric sepsis depend on the clinical presentation, time elapsed since identification and context. The RED strategy underscores the idea that these interventions are dynamic, not static, and are tailored to the course of the disease (precision medicine) and the available resources. CRRT, continuous renal replacement therapy.

The RED strategy phases include (Figure 1).

## 1 Resuscitation

*James, a previously healthy six-year-old boy, presents to the emergency room with signs of septic shock, including hypotension, tachycardia, and cold extremities. Antibiotics are started within the first hour of care, and his blood pressure improves slightly after the initial fluid bolus; however, hypotension persists, raising the dilemma of whether to continue fluid resuscitation or start vasopressors to avoid fluid overload. The team decides to administer a second fluid bolus and, given the suboptimal response, initiates epinephrine while considering a transfer to the pediatric intensive care unit (PICU) to continue treatment. The PICU informs the team that there are no immediately available beds.*

The main goal of this phase is fluid resuscitation, seeking to optimize both macrocirculatory parameters (cardiac output and/or arterial blood pressure) and microcirculatory parameters (tissue perfusion and oxygenation). Streamlined fluid resuscitation and early initiation of vasoactive drugs are becoming more common in the initial management of pediatric sepsis and septic shock (13, 14). Although the use of crystalloid boluses in sepsis resuscitation has historically been considered a cornerstone treatment, this strategy is not free of adverse effects. However, despite these limitations, timely fluid resuscitation in children with sepsis is a universally accepted strategy used in almost all possible care settings.

### 1.1 Fluid therapy

Fluid resuscitation is used to correct the actual and relative hypovolemia caused by decreased fluid intake prior to presentation, increased insensible losses, vasodilation, and increased capillary leak. The most recent pediatric sepsis management guidelines recommend applying fluid boluses according to the care context and the patient's clinical condition (3, 4). All hypotensive children, regardless of the availability of resources, should receive balanced crystalloid boluses at 10–20 ml/kg/dose within the first hour of care (3). A rapid administration of crystalloid loads has been associated with greater endothelial injury, shock, and respiratory distress, while slower administration has been associated with little or transient cardiac output recovery (15–17). Studies are needed in children to help clarify the most effective fluid bolus administration rate according to the context, phenotype and severity of presentation. For normotensive patients with hypoperfusion (prolonged capillary refill, altered consciousness), a crystalloid bolus is only recommended when critical care services are available. The *Surviving Sepsis Campaign* (SSC) guidelines recommend only using maintenance fluids, without crystalloid boluses, if critical care services or support are not available (3, 4). However, this recommendation should be integrated into the context and capacity of the care setting. A patient may be severely dehydrated, hypoperfused and require a fluid bolus despite the lack of available critical care support. This is an example of how each sepsis intervention should be aimed at personalization. Recently, the Fluid Resuscitation for Suspected Septic shock in Paediatric Emergency Departments (FRESSPED) study evaluated the adherence to SSC guidelines in the pediatric emergency rooms of various hospitals (18). The results showed high adherence at the beginning of fluid resuscitation but moderate adherence to the volume and type of crystalloids used. The main barriers reported by physicians were difficult venous access, lack of team training and missing or outdated protocols.

An important aspect to keep in mind is that improvements in cardiac output after fluid boluses in children tend to be transient. Long et al. (19) found an increased cardiac index in 63% of patients five minutes after infusing crystalloid boluses, which decreased to 14% after 60 min. Suchitra et al. (20) found that the hemodynamic response to a fluid bolus was unpredictable in children with sepsis. Patients tended to have an improvement in mean arterial pressure (MAP) but not necessarily increased cardiac output after a fluid bolus. In fact, in some patients, fluid boluses were associated with a vasodilating effect, and those who did not experience MAP recovery after a crystalloid bolus had greater mortality (20). Rapid fluid redistribution and excretion in children explains why up to 50% of the infused crystalloid volume may leave the intravascular space within the first 30 min, with significantly higher urinary excretion than in adults (21). This physiological characteristic underscores the importance of dynamic management in pediatrics, adjusting fluid resuscitation to maintain perfusion without causing hypervolemia.

In patients with sepsis, the fluid redistribution mechanism is influenced by several pathophysiological factors like the degree of endothelial dysfunction, cardiac output status, and inflammatory activation. Some patients may develop respiratory distress, greater oxygen requirements, intra-abdominal hypertension and/or acute kidney injury (AKI) after a fluid load, due to increased capillary leak and tissue edema. These patients have been called “*fluid intolerant*” (22). This low tolerance to fluid boluses could be explained by macrocirculatory dysfunction (heart failure) or worsening endothelial activation related to fluid loads, which some authors have termed resuscitation-associated endotheliopathy (RAsE). The RAsE concept suggests that endothelial activation and macrocirculatory dysfunction contribute to low fluid tolerance, which limits the effectiveness of crystalloids in some patients. Therefore, not all patients are simply “*fluid responders*” or “*nonresponders*,” but rather may have a more complex combination of factors that affect their response to fluid treatment (17, 23). One of these factors is sympathoadrenal hyperactivation related to endothelial activation, glycocalyx injury and altered perfusion, a phenomenon known as shock-induced endotheliopathy (SHINE) (24).

#### Pathophysiological aspects

##### a. Macrocirculation

**Figure 2:**
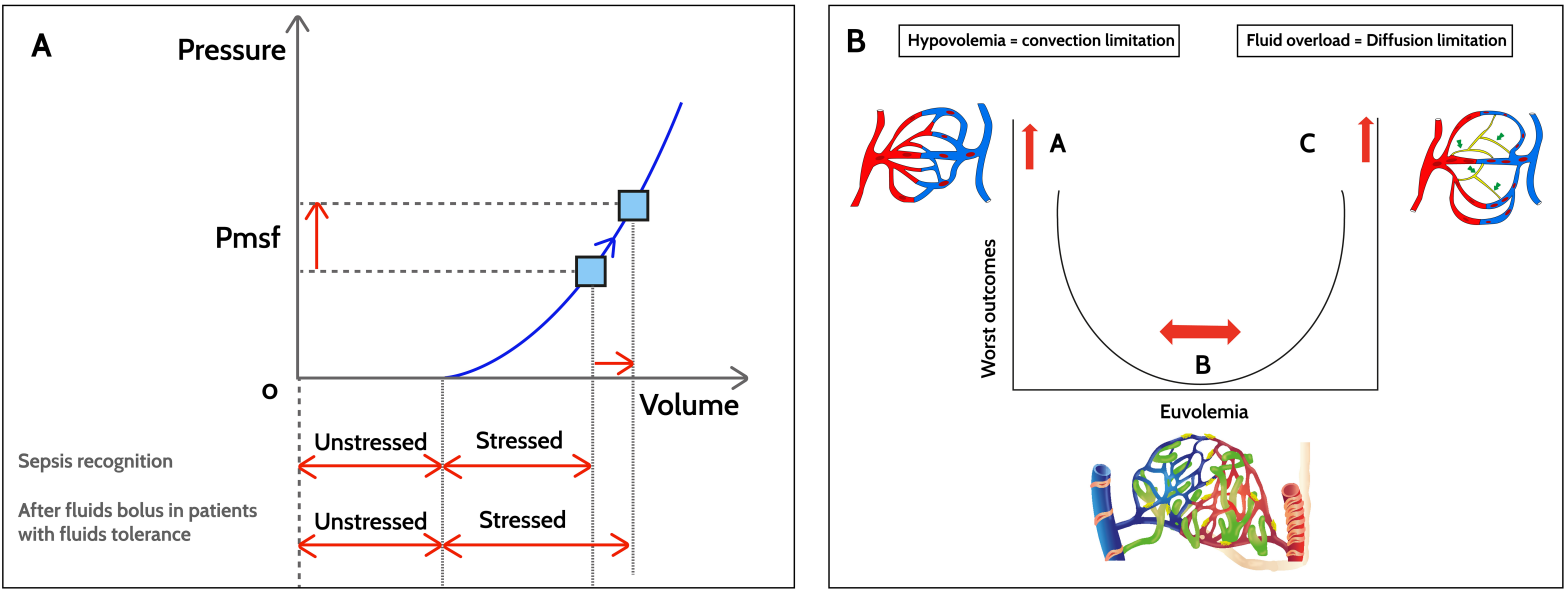
Hemodynamic changes after fluid boluses in sepsis. **(A)** *Macrocirculation*. After fluid boluses, mean systemic filling pressure (*Pmsf*) increases due to increased stressed volume, with the unstressed volume remaining constant. **(B)** *Microcirculation*. In hypovolemia (point A), there is an abnormal driving pressure (DP) that determines the convective flow. The DP results from subtracting the venule blood pressure from the precapillary blood pressure. Point B corresponds to euvolemia without microvascular abnormalities with a lower risk of worse outcomes. In fluid overload with tissue edema, diffusive flow is altered (point C). The gas exchange distance increases and, due to microvascular heterogeneity, the functional capillary density (number of perfused capillaries/tissue area) decreases in sepsis.

The hemodynamic response to fluid boluses in children with sepsis is associated with both macro- and microcirculatory changes (Figure 2). The first change is expanded intravascular volume. According to Guyton et al. (25), intravascular volume can be divided into stressed and unstressed volume. Stressed volume is that which distends the blood vessel walls with a simultaneous increase in pressure, while unstressed volume fills the blood vessels but does not generate any pressure. A 10–20 ml/kg fluid bolus temporarily increases the stressed volume, thereby increasing the mean systemic filling pressure (*Pmsf*), which is the pressure in the vessels without blood flow or during circulatory arrest (Figure 2A) (26). However, the hemodynamic response to fluid boluses varies in pediatric septic shock, with evidence of no increase in ejection volume with a fluid challenge (despite an increased *Pmsf*) and even a decrease in blood pressure in some cases (26).

Similarly, animal models of septic shock have shown that recovery of the macrocirculatory variables with fluid boluses is not necessarily associated with improved microvascular flow and oxygen delivery to the tissues (27). This loss of *hemodynamic coherence* has been associated with worse outcomes and greater mortality (28). In observational studies in adults, improved microvascular blood flow after a fluid bolus has been found to occur only in the first 48 h after identifying sepsis (29). Persistent microcirculatory dysfunction, especially low 4–6-micron capillary density (known as *functional capillary density*), in children with sepsis after fluid boluses was found to be associated with greater mortality (17% vs. 6%) and worse outcomes, despite normalized macrocirculatory variables, when compared to children with sepsis and a normal functional capillary density (30).

##### b. Microcirculation

It has been generally accepted that normalization of tissue perfusion and oxygen delivery are the ultimate endpoints for fluid resuscitation in septic shock. Microcirculation changes after fluid boluses are largely determined by the timing of the interventions and the extent macrocirculatory abnormalities. Oxygen is transported in the microcirculation through convection and diffusion (Figure 2B). Convection depends on the microcirculatory blood flow (determined by the arteriolar tone) and the oxygen content (which depends on the capillary hematocrit). Diffusion depends on the exchange distance (greater in tissue edema), the capillary/mitochondrial partial oxygen pressure (PO_2_) gradient and, finally, the gas exchange area. Under normal conditions, only 25%–30% of the capillaries are perfused, and the cardiovascular system is extremely efficient in adjusting blood flow to the metabolic demands of the tissues and recruiting additional capillaries when necessary (31). This ensures tissue perfusion without a high metabolic cost.

Microcirculatory changes during sepsis entail heterogeneity in capillary perfusion, with slow-flow areas (approximately 100 µm/s) and others with normal flow (400–500 µm/s) (32). Additionally, there is a lower density of vessels smaller than 10 µm, reducing the functional capacity of the microcirculation (29). The red blood cell velocity in the perfused vessels does not change according to the width of the vessel but is influenced by the velocity of the larger capillaries, which suggests that small capillaries (4–6 µm) do not respond appropriately to local changes in oxygen demand, which translates into clinical perfusion alterations (30). In patients with septic shock, the disassociation between tissue oxygen demand and vascular perfusion is thought to be responsible for the progression to multiple organ dysfunction (MODS) (28, 31).

Mitochondrial dysfunction is one of the most important consequences of this oxygen delivery imbalance in the cells. Under normal conditions, mitochondria use approximately 98% of the available cellular oxygen for energy production through the Krebs cycle. Mitochondrial dysfunction in sepsis is associated with the onset and severity of MODS (33). Interventions aimed at improving mitochondrial activity with medications (thiamine) or micronutrients (ascorbic acid, tocopherol, selenium and zinc) have been termed “*metabolic resuscitation*” (34). Although these interventions have theoretical benefits, they do not have enough evidence yet to support their widespread use. Genomic, metabolomic and pharmacogenomic development is expected to identify the specific groups of patients who would benefit from the recovery of mitochondrial function with these pharmacological measures.

After crystalloid boluses, there are changes in the capillary *driving pressure* (the difference between precapillary and venule pressure) with improved convection, and changes in diffusion with more recruitment of capillaries and better functional capillary density. However, these responses to fluid boluses have been seen in adults only in the 48 h after sepsis diagnosis (29). Pranskunas et al. reported that patients who had improved microcirculation perfusion after fluid boluses had an associated improvement in organ function (35). Furthermore, in children, unbalanced fluid boluses have been associated with negative microcirculatory changes, including glycocalyx degradation and increased endothelial permeability (36). In this regard, the volume of intravenous fluids administered during sepsis resuscitation in adults has been found to be independently associated with the degree of glycocalyx degradation (37). This layer, that covers the endothelial cells, is essential for microvascular homeostasis, mediates the vasorelaxation induced by shear stress and prevents leukocyte adhesion to the endothelial cells. In sepsis, tumor necrosis factor-*α* and angiopoietin-2, among others, induce heparanase expression and activation, which causes endothelial dysfunction and organ insult mediated by damage to heparan sulfate, a component of the endothelial glycocalyx (38). Heparanase and the inflammatory response in sepsis also cause degradation of syndecan-1, another structural component of the glycocalyx. These phenomena lead to the loss of integrity of the protective layer of the endothelial cell, increase microvascular permeability and foster the onset of capillary leak syndrome.

### 1.2 Vasoactive medications

In this initial resuscitation phase of the dynamic strategy it may be necessary to begin vasoactive support. Pediatric sepsis guidelines (3, 4) recommend initiating vasoactive support when signs of hypoperfusion persist after fluid resuscitation or signs of fluid overload appear. The SSC recommends considering beginning vasoactive drugs after 40–60 ml/kg of crystalloid boluses. However, a recent multicenter randomized pilot trial comparing early initiation of adrenaline (after a 20 ml/kg crystalloid bolus) vs. the treatment recommended by SSC found that there was a lower total 24-hour fluid input in the intervention group, with no differences in the frequency of organ dysfunction, pediatric intensive care unit (PICU) admission or length of PICU stay (13). Another open-label trial in children with sepsis found that early initiation of adrenaline (after 40 ml/kg of crystalloids) reduced the need for mechanical ventilation, as well as persistent shock and mortality (39). In adults, observational studies have shown that early administration of noradrenaline (less than one hour after identifying shock) has been associated with a reduction in the total volume of fluids administered and lower 28-day mortality (40, 41).

There are no studies in children specifically comparing adrenaline (or epinephrine) with noradrenaline (or norepinephrine) as a first-line vasoactive drug in septic shock. Banothu KK et al. (42) conducted an open-label randomized controlled study at a single center in India, comparing the effectiveness of two treatment regimens in children with fluid-refractory septic shock. Two approaches were studied: norepinephrine plus dobutamine vs. epinephrine as a first-line vasoactive agent. The primary objective was to determine which of these treatments offered better outcomes in terms of hemodynamic stabilization and reduced mortality. The results showed that both approaches were effective for managing shock. However, there were differences in their side effect profiles and the time required to recover cardiovascular function, with the norepinephrine plus dobutamine group resolving shock more rapidly (HR 1.84; 95% CI 1.11–3.08).

When there is evidence of low cardiac output, clinicians prefer adrenaline or dobutamine, and when there is evidence of vasodilation, noradrenaline is preferred. Both drugs stimulate the *beta* 1 adrenergic receptors, with increased chronotropy and inotropy, and the alpha-adrenergic receptors, with increased peripheral vascular resistance (PVR) (10). By increasing the PVR, some vasopressors also increase venous tone, increasing *Pmsf* and adding to the effect of the crystalloid boluses (40).

## 2 Equilibrium

*James develops respiratory failure, is intubated in the emergency room, and an x-ray shows signs of pneumonia. The addition of peripherally administered noradrenaline is necessary to maintain the target blood pressure. He is admitted to intensive care and the team begins invasive monitoring and places a central venous catheter, but prolonged capillary refill persists despite achieving the macrocirculation goals. In light of the persistent signs of hypoperfusion despite fluid resuscitation and vasoactive drugs, the team decides to begin an inodilator.*

The goal of this phase is to maintain a hemodynamic balance in both the macro and microcirculation after the initial fluid bolus and vasoactive support interventions. It often occurs within a few hours of sepsis diagnosis. In this phase of hemodynamic management, it is important to adjust the vasoactive drugs and titrate fluid input to avoid unnecessary additional crystalloid boluses, which can lead to fluid overload and worse outcomes (43, 44).

### 2.1 Objectifying the need for additional fluid boluses

Identifying children in septic shock who could benefit from additional crystalloid boluses tends to be a significant clinical challenge. According to the availability of resources, clinical assessments and minimally invasive monitoring tools have been used to determine the fluid response status in critically ill patients (Table 1). A systematic review and meta-analysis of 62 pediatric studies that sought to evaluate the performance of different tools in predicting response to fluids in critically ill children found that the variables with a good capacity for predicting the response to fluids were passive leg raising stroke volume (PLR-SV), respiratory variation in aortic peak flow (RVAF), and left ventricular velocity time integral (LVVTI) measured using an ultrasound device (45). However, these tools are often not available at the bedside. Furthermore, the association between preload recovery as defined by ultrasound techniques and actual clinical improvement is unclear and requires further study. When these tools are not available, tissue perfusion monitoring (i.e., capillary refill time) can guide the clinician on the risks or benefits of administering additional fluid boluses. A post-hoc analysis of the ANDROMEDA-SHOCK trial (which included a systematic evaluation of the baseline response to fluids prior to beginning the protocol) found that, in a significant percentage of patients the fluid resuscitation could be guided by clinical variables like capillary refill time (46). In patients who did not respond to fluid resuscitation, fluid boluses could be stopped with no negative impact on the relevant clinical outcomes.

### 2.2 Monitoring fluid creep

Another important aspect in all the hemodynamic intervention phases, especially in this equilibrium phase, is to consider the volume administered that is not related to fluid boluses. The amount of maintenance fluids, continuous infusions, nutrition, blood products, medication dilution fluids, and flushes to maintain the patency of intravascular lines can contribute to hypervolemia in the post-resuscitation phase. The contribution of these non-resuscitation fluids to fluid overload has been termed “*fluid creep*” (47, 48). Some studies have found that fluid creep accounts for a third of the total daily administered fluid, with its proportion gradually increasing throughout the PICU stay, becoming the main source of fluids by the fourth or fifth day of PICU stay (41). Barhight et al. (6) evaluated 14,483 PICU patients in two hospitals and found that more than half of these children received non-resuscitation fluid beyond hydration requirements, which was associated with greater mortality (a 1% increase in mortality for every 10 ml/kg of excess fluid) regardless of age, Pediatric Risk of Mortality III score, study site, acute kidney injury, resuscitation volume and volume output. Excess maintenance fluids are a modifiable factor that can contribute to hypervolemia and should be actively titrated, particularly in the post-resuscitation phase. Performing proper daily fluid balance monitoring, tracking inputs and outputs along with the patient's weight, can help the clinician prevent overhydration and adverse outcomes which have been related to hypervolemia (AKI, abdominal hypertension or greater mortality).

## 3 De-escalation

*James is stabilized, but after 48 h of care, he has a positive balance of 22% of his body weight, with significant generalized edema, and he develops oliguria and mild azotemia. The team decides to begin loop diuretics after confirming that James is on low doses of vasoactives and is hemodynamically stable.*

After the initial stabilization and reaching equilibrium, the clinician should concentrate on gradually decreasing the hemodynamic support, limiting exposure to unnecessary fluids and facilitating the removal of excess fluids. During the resuscitation and equilibrium phases there is often hypervolemia, positive balances and soft tissue edema due to fluid administration often complicated by AKI and increased endothelial permeability with fluid transfer from the intravascular to the interstitial space.

### Pathophysiological aspects

Under normal conditions, there is a close interaction between microcirculation and the interstitial extracellular matrix. The integrity of the endothelial barrier, the glycocalyx layer and interstitial pressure help regulate transcapillary flow between the intravascular and interstitial spaces (6). Interstitial space pressure is kept within a narrow range (between −2 and −3 mmHg) by the constant tension exerted by the fibroblasts on the collagen bundles through the *B*-1 integrin transmembrane protein (49, 50). This tension, coupled with appropriate functioning of the lymphatic system, is essential for keeping the interstitial space free of excess fluid (51). Under inflammatory conditions, increased cytokines (mainly tumor necrosis factor alpha, interleukin-1B, and interleukin-6) and matrix proteases result in a loss of binding between the *B*1 integrins and collagen fibers (52). Furthermore, the endothelial activation, glycocalyx damage, loss of intercellular binding and lymphatic system saturation that occur in patients with sepsis lead to increased filtration pressure (capillary pressure – interstitial pressure) with subsequent fluid accumulation in the interstitial space (51, 52). Under inflammatory conditions, the interstitial pressure has been found to reach up to −100 mmHg, which progressively increases the amount of fluid accumulated in the interstitium, a phenomenon that has been called *interstitial suction* (53). The clinical expression of this condition is tissue edema with hypoperfusion and associated organ failure, often found in children with capillary leak and septic shock.

### 3.1 Active fluid removal

One way to reduce hypervolemia, sustain euvolemia and optimize tissue perfusion is through active fluid removal. Very often, the treatment measures used to decrease hypervolemia are not planned and can lead to relative hypovolemia and new, unnecessary fluid boluses. A survey by Aramburo et al. (5) in 48 countries showed that 93% of physicians employed active fluid removal or fluid limiting practices for children in critical care. The most common interventions were the use of loop diuretics (93.3%), restriction or avoidance of maintenance fluids (86.6%), minimizing drug diluents (72.4%) and the use of renal replacement therapy to prevent or treat fluid accumulation (55%), especially in children with poor response to diuretics or evidence of severe AKI. In adults, active fluid removal has been associated with a reduction in the duration of mechanical ventilation, shorter ICU length of stay and lower mortality (54).

Another active fluid removal strategy employed commonly is the use of hyperoncotic albumin (20 or 25% albumin fluid) in conjunction with the diuretics. In adults being ventilated due to lung injury, the use of hyperoncotic albumin with furosemide, coupled with adjusted positive end-expiratory pressure, has been associated with a negative cumulative fluid balance and decreased lung water (55). Following initial resuscitation in adults with sepsis, hyperoncotic albumin has been associated with improved tissue hypoperfusion compared to 0.9% saline solution (55). In patients with sepsis, plasma and albumin have also been found to have a potential protective effect on the endothelium through antioxidant and anti-inflammatory effects (56, 57). Likewise, in children with sepsis, the correction of hypoalbuminemia has been associated with improved functional capillary density, endothelial glycocalyx damage recovery and lower levels of *angiopoietin*-2 (58, 59). In addition, a multicenter observational study of children with a sepsis phenotype characterized by persistent hypoxemia, encephalopathy and shock -which is associated with increased systemic inflammation and endothelial activation- found that those who received 0.5 g/kg or more of intravenous albumin within the first 24 h of care were associated with a higher survival rate (75% vs. 66%) than those who did not after adjusting for confounders (60).

### 3.2 Renal replacement therapy

Another strategy used to remove fluids is renal replacement therapy (RRT). Acute kidney injury is common in children with sepsis and may require extracorporeal renal support therapies when there is no response to diuretics (2, 4). In adults with sepsis, there have been conflicting study results regarding the use of these therapies to remove excess fluids (61). No differences have been found in mortality, length of ICU stay or duration of AKI with early vs. late RRT (62). In a recent multinational survey, 55% of the physicians reported using RRT to prevent or treat hypervolemia in critically ill children (6). In this phase of the RED strategy, one of the most important aspects is AKI prevention, avoiding overhydration or high doses of vasopressors. A recent systematic review and meta-analysis in children found that a fluid overload greater than 10% at any time during the PICU stay was associated with a greater need for mechanical ventilation and mortality (44).

### 3.3 Tissue perfusion monitoring

Active fluid removal must be closely monitored and individually adjusted to each case. The prerequisite for active fluid removal is an achievement of hemodynamic stability with resolved hypoperfusion and requirement for low (or no) doses of vasoactive drugs. Furthermore, close tracking of fluid balance (as well as daily weights when possible) is needed estimate the amount of accumulated fluid. Close monitoring of serum lactate and capillary refill time is useful for guiding this fluid redistribution phase and can help determine the microcirculation status of children with sepsis (46, 63). Pediatric randomized trials are needed to evaluate the best strategy for performing active fluid removal and the most appropriate monitoring tools (64).

## Limitations

The RED strategy proposal has not yet been standardized or validated in clinical studies. It clusters a series of updated interventions for hemodynamic management in sepsis which should be evaluated in prospective studies. We do not know if reaching hemodynamic goals will translate into better neurological and functional outcomes. In addition, the varied hemodynamic response in the different pediatric sepsis phenotypes and the challenges to clinical implementation in different care settings are aspects that must be evaluated in the RED strategy. However, the RED strategy brings a more dynamic and practical perspective to the circulatory management of pediatric shock, by unifying the optimization and stabilization phases (from the ROSE strategy for adults) under the concept of “*equilibrium*.” This better reflects the clinical reality, where transitions between these phases are often blurred, and continuous and adaptable management is needed to achieve homeostasis without affecting perfusion or tissue oxygenation. Moreover, by including the “de-escalation” phase as an explicit component, RED addresses the growing evidence of the importance of minimizing hypervolemia and withdrawing hemodynamic support in a controlled fashion, which is associated with better clinical outcomes (44, 54, 63). This simpler, action-oriented framework facilitates implementation in pediatric scenarios, especially in settings with limited resources.

## Conclusion

Sepsis is one of the main causes of morbidity, mortality and new functional disorders in children worldwide. The cardiovascular system is one of the most frequently affected, with both macro- and microcirculation abnormalities. Fluid resuscitation and vasoactive drugs modify the clinical course of the disease but are not free of adverse effects. The structured and personalized use of these interventions during resuscitation, the rational administration of non-resuscitation fluids, and the timely removal of accumulated fluid have the potential to improve outcomes in such a complex and dynamic syndrome. The proposed RED strategy provides a holistic, phased approach to the hemodynamic management of children with circulatory involvement, anticipates potential complications associated with these interventions, and aims at faster cardiovascular stabilization and improved clinical outcomes.

## References

[1] Rudd KE Johnson SC Agesa KM Shackelford KA Tsoi D Kievlan DR Global, regional, and national sepsis incidence and mortality, 1990–2017: analysis for the global burden of disease study. Lancet. (2020) 395(10219):200–11. 10.1016/S0140-6736(19)32989-7 31954465 PMC6970225

[2] Weiss SL Balamuth F Hensley J Fitzgerald JC Bush J Nadkarni VM The epidemiology of hospital death following pediatric severe sepsis: when, why, and how children with sepsis die. Pediatr Crit Care Med. (2017) 18(9):823–30. 10.1097/PCC.0000000000001222 28549024 PMC5581233

[3] Weiss SL Peters MJ Alhazzani W Agus MSD Flori HR Inwald DP Surviving sepsis campaign international guidelines for the management of septic shock and sepsis-associated organ dysfunction in children. Pediatr Crit Care Med. (2020) 21(2):e52–e106. 10.1097/PCC.0000000000002198 32032273

[4] Fernández-Sarmiento J De Souza DC Martinez A Nieto V López-Herce J Soares Lanziotti V Latin American consensus on the management of sepsis in children: sociedad latinoamericana de cuidados intensivos pediátricos [Latin American pediatric intensive care society] (SLACIP) task force: executive summary. J Intensive Care Med. (2022) 37(6):753–63. 10.1177/08850666211054444 34812664

[5] Aramburo A Raman S Silversides JA Schlapbach LJ Gibbons KS Ramnarayan P Fluid management and active fluid removal practices: a global survey of paediatric critical care physicians. Intensive Care Med Paediatr Neonatal. (2024) 2(16):1–10. 10.1007/s44253-024-00038-1

[6] Barhight MF Nelson D Chong G Basu RK Sanchez-Pinto LN. Non-resuscitation fluid in excess of hydration requirements is associated with higher mortality in critically ill children. Pediatr Res. (2022) 91(1):235–40. 10.1038/s41390-021-01456-z 33731814 PMC7968408

[7] Lintz VC Vieira RA Carioca FL Ferraz IS Silva HM Ventura AMC Fluid accumulation in critically ill children: a systematic review and meta-analysis. EClinicalMedicine. (2024) 74:102714. 10.1016/j.eclinm.2024.102714 39070177 PMC11278930

[8] Malbrain M Van Regenmortel N Saugel B De Tavernier B Van Gaal PJ Joannes-Boyau O Principles of fluid management and stewardship in septic shock: it is time to consider the four d’s and the four phases of fluid therapy. Ann Intensive Care. (2018) 8(1):6. 10.1186/s13613-018-0402-x 29789983 PMC5964054

[9] Vincent JL van der Poll T Marshall JC. The end of “one size fits all” sepsis therapies: toward an individualized approach. Biomedicines. (2022) 10(9):2260. 10.3390/biomedicines10092260 36140361 PMC9496597

[10] Vincent JL De Backer D. Circulatory shock. N Engl J Med. (2013) 369:1726–34. 10.1056/NEJMra1208943 24171518

[11] Monnet X Lai C Teboul JL. How I personalize fluid therapy in septic shock? Crit Care. (2023) 27(1):123. 10.1186/s13054-023-04363-3 36964573 PMC10039545

[12] Hagedoorn NN Zachariasse JM Moll HA. Association between hypotension and serious illness in the emergency department: an observational study. Arch Dis Child. (2020) 105(6):545–51. 10.1136/archdischild-2018-316231 30948363 PMC7285787

[13] Harley A George S Phillips N King M Long D Keijzers G Resuscitation with early adrenaline infusion for children with septic shock: a randomized pilot trial. Ped Crit Care Med. (2024) 25(2):106–17. 10.1097/PCC.0000000000003351 PMC10798589 38240535

[14] Obonyo NG Olupot-Olupot P Mpoya A Nitziyaremye J Chebet M Uyoga S A clinical and physiological prospective observational study on the management of pediatric shock in the post-fluid expansion as supportive therapy trial era. Ped Crit Care Med. (2023) 23(7):502–13. 10.1097/PCC.0000000000002968 PMC7613033 35446796

[15] Mullan PC Pruitt CM Levasseur KA Macias CG Paul R Depinet H Intravenous fluid bolus rates associated with outcomes in pediatric sepsis: a multi-center analysis. Open Access Emerg Med. (2022) 14:375–84. 10.2147/OAEM.S368442 35924031 PMC9342868

[16] Sankar J Ismail J Sankar MJ Suresh CP Meena RS. Fluid bolus over 15–20 versus 5–10 min each in the first hour of resuscitation in children with septic shock: a randomized controlled trial. Pediatr Crit Care Med. (2017) 18(10):e435–45. 10.1097/PCC.0000000000001269 28777139

[17] Obonyo NG Sela DP Raman S Rachakonda R Schneider B Hoe LES Resuscitation-associated endotheliopathy (RAsE): a conceptual framework based on a systematic review and meta-analysis. Syst Rev. (2023) 12(1):221. 10.1186/s13643-023-02385-0 37990333 PMC10664580

[18] San Geroteo J Levy M Bailhache M De Jorna C Privat E Gasmi O Assessment of adherence to the 2020 surviving sepsis campaign guidelines for fluid resuscitation in children with suspected septic shock in paediatric emergency departments: a prospective multicentre study. Arch Dis Child. (2024) 109(8):636–41. 10.1136/archdischild-2023-325837 38499323

[19] Long E Babl FE Oakley E Sheridan B Duke T. Pediatric research in emergency departments international collaborative (PREDICT). Cardiac index changes with fluid bolus therapy in children with sepsis-an observational study. Pediatr Crit Care Med. (2018) 19(6):513–8. 10.1097/PCC.0000000000001534 29533353

[20] Ranjit S Natraj R Kissoon N Thiagarajan RR Ramakrishnan B Monge García MI. Variability in the hemodynamic response to fluid bolus in pediatric septic shock. Pediatr Crit Care Med. (2021) 22(8):e448–58. 10.1097/PCC.0000000000002714 33750093

[21] Li Y Hahn RG Hu Y Xiang Y Zhu S. Plasma and renal clearances of lactated ringer’s solution in pediatric and adult patients just before anesthesia is induced. Paediatr Anaesth. (2009) 19(7):682–7. 10.1111/j.1460-9592.2009.03047.x 19638113

[22] Kattan E Castro R Miralles-Aguiar F Hernández G Rola P. The emerging concept of fluid tolerance: a position paper. J Crit Care. (2022) 71:154070. 10.1016/j.jcrc.2022.154070 35660844

[23] Trigkidis KK Routsi C Kokkoris S. Correlation of venous excess ultrasound (VExUS) score to fluid responsiveness in critically ill patients. J Crit Care. (2024) 7(85):154905. 10.1016/j.jcrc.2024.154905 39244804

[24] Johansson PI Stensballe J Ostrowski SR. Shock induced endotheliopathy (SHINE) in acute critical illness - a unifying pathophysiologic mechanism. Crit Care. (2017) 21(1):25. 10.1186/s13054-017-1605-5 28179016 PMC5299749

[25] Guyton AC Polizo D Armstrong GG. Mean circulatory filling pressure measured immediately after cessation of heart pumping. Am J Physiol. (1954) 179:261–7. 10.1152/ajplegacy.1954.179.2.261 13218155

[26] Aya HD Rhodes A Fletcher N Grounds RM Cecconi M. Transient stop-flow arm arterial-venous equilibrium pressure measurement: determination of precision of the technique. J Clin Monit Comput. (2016) 30(1):55–61. 10.1007/s10877-015-9682-y 25749976

[27] Can I Egbert G. Microcirculatory and mitocondrial hypoxia in sepsis, shock and resuscitation. J Appl Physiol. (2016) 120:226–35. 10.24875/BMHIM.20000323 26066826

[28] Can I. Hemodynamic coherence and the rationale for monitoring the microcirculation. Crit Care. (2015) 19:3. 10.1186/cc14726 26729241 PMC4699073

[29] Ospina-Tascon G Neves AP Occhipinti G Donadello K Buchele G Simion D Effects of fluids on microvascular perfusion in patients with severe sepsis. Intensive Care Med. (2010) 36(6):949–55. 10.1007/s00134-010-1843-3 20221744

[30] Fernández-Sarmiento J Lamprea S Barrera S Acevedo L Duque C Trujillo M The association between prolonged capillary refill time and microcirculation changes in children with sepsis. BMC Pediatr. (2024) 24(68):1–10. 10.1186/s12887-024-04524-5 38245695 PMC10799439

[31] Roy TK Secomb TW. Functional implications of microvascular heterogeneity for oxygen uptake and utilization. Physiol Rep. (2022) 10(10):e15303. 10.14814/phy2.15303 35581743 PMC9114652

[32] Yajnik V Maarouf R. Sepsis and the microcirculation: the impact on outcomes. Curr Opin Anaesthesiol. (2022) 35(2):230–5. 10.1097/ACO.0000000000001098 35081058

[33] Leite HP de Lima LF. Metabolic resuscitation in sepsis: a necessary step beyond the hemodynamic? J Thorac Dis. (2016) 8(7):E552–7. 10.21037/jtd.2016.05.37 27501325 PMC4958886

[34] Donnino MW Andersen LW Chase M Berg KM Tidswell M Giberson T Randomized, double-blind, placebo-controlled trial of thiamine as a metabolic resuscitator in septic shock: a pilot study. Crit Care Med. (2016) 44:360–7. 10.1097/CCM.0000000000001572 26771781 PMC4754670

[35] Pranskunas A Koopmans M Koetsier PM Pilvinis V Boerma EC. Microcirculatory blood flow as a tool to select ICU patients eligible for fluid therapy. Intensive Care Med. (2013) 39(4):612–9. 10.1007/s00134-012-2793-8 23263029 PMC3607718

[36] Fernández-Sarmiento J Salazar-Peláez LM Acevedo L Niño-Serna LF Flórez S Alarcón-Forero L Endothelial and glycocalyx biomarkers in children with sepsis after one bolus of unbalanced or balanced crystalloids. Pediatr Crit Care Med. (2023) 24(3):213–21. 10.1097/PCC.0000000000003123 36598246

[37] Hippensteel JA Uchimido R Tyler PD Burke RC Han X Zhang F Intravenous fluid resuscitation is associated with septic endothelial glycocalyx degradation. Crit Care. (2019) 23(1):259. 10.1186/s13054-019-2534-2 31337421 PMC6652002

[38] Schmidt EP Yang Y Janssen WJ Gandjeva A Perez MJ Barthel L The pulmonary endothelial glycocalyx regulates neutrophil adhesion and lung injury during experimental sepsis. Nat Med. (2012) 18(8):1217–23. 10.1038/nm.2843 22820644 PMC3723751

[39] Iramain R Ortiz J Jara A Bogado N Morinigio R Cardozo L Fluid resuscitation and inotropic support in patients with septic shock treated in pediatric emergency department: an open-label trial. Cureus. (2022) 14(10):e30029. 10.7759/cureus.30029 36225249 PMC9541896

[40] Ospina-Tascon GA Hernandez G Alvarez I Calderon-Tapia LE Manzano-Nunez R Sanchez-Ortiz AI Effects of very early start of norepinephrine in patients with septic shock: a propensity score-based analysis. Crit Care. (2020) 24(1):52. 10.1186/s13054-020-2756-3 32059682 PMC7023737

[41] Xu F Zhong R Shi S Zeng Y Tang Z. Early initiation of norepinephrine in patients with septic shock: a propensity score-based analysis. Am J Emerg Med. (2022) 54:287–96. 10.1016/j.ajem.2022.01.063 35227959

[42] Banothu KK Sankar J Kumar UV Gupta P Pathak M Jat KR A randomized controlled trial of norepinephrine plus dobutamine versus epinephrine as first-line vasoactive agents in children with fluid refractory cold septic shock. Crit Care Explor. (2022) 5(1):e0815. 10.1097/CCE.0000000000000815 36600781 PMC9799172

[43] Ranjit S Kissoon N Argent A Inwald D Ventura AMC Jaborinsky R Haemodynamic support for paediatric septic shock: a global perspective. Lancet Child Adolesc Health. (2023) 7(8):588–98. 10.1016/S2352-4642(23)00103-7 37354910

[44] Fernández-Sarmiento J Sierra-Zuñiga MF Salazar González MP Lucena N Soares Lanziotti V Agudelo S. Association between fluid overload and mortality in children with sepsis: a systematic review and meta-analysis. BMJ Paediatr Open. (2023) 7(1):e002094. 10.1136/bmjpo-2023-002094 37989355 PMC10668252

[45] Walker SB Winters JM Schauer JM Murphy P Fawcett A Sanchez-Pinto LN. Performance of tools and measures to predict fluid responsiveness in pediatric shock and critical illness: a systematic review and meta-analysis. Pediatr Crit Care Med. (2024) 25(1):24–36. 10.1097/PCC.0000000000003320 37462437 PMC10794582

[46] Kattan E Ospina-Tascón GA Teboul JL Castro R Cecconi M Ferri G Systematic assessment of fluid responsiveness during early septic shock resuscitation: secondary analysis of the ANDROMEDA-SHOCK trial. Crit Care. (2020) 24(1):23. 10.1186/s13054-020-2732-y 31973735 PMC6979284

[47] Van Regenmortel N Verbrugghe W Roelant E Van den Wyngaert T Jorens PG. Maintenance fluid therapy and fluid creep impose more significant fluid, sodium, and chloride burdens than resuscitation fluids in critically ill patients: a retrospective study in a tertiary mixed ICU population. Intensive Care Med. (2018) 44(4):409–17. 10.1007/s00134-018-5147-3 29589054 PMC5924672

[48] Brossier DW Tume LN Briant AR Jotterand Chaparro C Moullet C Rooze S. ESPNIC clinical practice guidelines: intravenous maintenance fluid therapy in acute and critically ill children- a systematic review and meta-analysis. Intensive Care Med. (2022) 48(12):1691–708. 10.1007/s00134-022-06882-z 36289081 PMC9705511

[49] Wiig H Reed RK Aukland K. Measurement if interstitial fluid pressure in dogs; evaluation of methods. Am J Physiol Heart Circ Physiol. (1987) 253:H283–90. 10.1152/ajpheart.1987.253.2.H283 3618802

[50] Liden A Karlsen TV Guss B Reed RK Rubin K. Integrin can substitute for collagen-binding B 1-integrins *in vivo* to maintain a homeostatic interstitial fluid pressure. Exp Physiol. (2018) 103:629–34. 10.1113/EP086902 29524327 PMC5947675

[51] Doyle AD Nazari SS Yamada KM. Cell-extracellular matrix dynamics. Phys Biol. (2022) 19(2):1–15. 10.1088/1478-3975/ac4390 34911051 PMC8855216

[52] Nedrebo T Berg A Reed RK. Effect of tumor necrosis factor-alpha, IL-1beta, and IL-6 on interstitial fluid pressure in rat skin. Am J Physiol. (1999) 2(77):H1857–62. 10.1152/ajpheart.1999.277.5.H1857 10564140

[53] Dargent A Dumargne H Labruyère M Brezillon S Brassart-Pasco S Blot M Role of the interstitium during septic shock: a key to the understanding of fluid dynamics? J Intensive Care. (2023) 11(1):44. 10.1186/s40560-023-00694-z 37817235 PMC10565984

[54] Silversides JA Fitzgerald E Manickavasagam US Lapinsky SE Nisenbaum R Hemmings N Role of active De-resuscitation after resuscitation (RADAR) investigators. De-resuscitation of patients with iatrogenic fluid overload is associated with reduced mortality in critical illness. Crit Care Med. (2018) 46(10):1600–7. 10.1097/CCM.0000000000003276 29985214

[55] Cordemans C De Laet I Van Regenmortel N Schoonheydt K Dits H Martin G Aiming for a negative fluid balance in patients with acute lung injury and increased intra-abdominal pressure: a pilot study looking at the effects of PAL-treatment. Ann Intensive Care. (2012) 2(Suppl 1):S15. 10.1186/2110-5820-2-S1-S15 22873416 PMC3390296

[56] Aldecoa C Llau JV Nuvials X Artigas A. Role of albumin in the preservation of endothelial glycocalyx integrity and the microcirculation: a review. Ann Intensive Care. (2020) 10(1):85. 10.1186/s13613-020-00697-1 32572647 PMC7310051

[57] Kravitz MS Kattouf N Stewart IJ Ginde AA Schmidt EP Shapiro NI. Plasma for prevention and treatment of glycocalyx degradation in trauma and sepsis. Crit Care. (2024) 28(1):254. 10.1186/s13054-024-05026-7 39033135 PMC11265047

[58] Fernández-Sarmiento J Hernández-Sarmiento R Salazar MP Barrera S Castilla V Duque C. The association between hypoalbuminemia and microcirculation, endothelium, and glycocalyx disorders in children with sepsis. Microcirculation. (2023) 30(8):e12829. 10.1111/micc.12829 37639384

[59] Sanchez-Pinto LN Bennett TD Stroup EK Luo Y Atreya M Bubeck Wardenburg J Derivation, validation, and clinical relevance of a pediatric sepsis phenotype with persistent hypoxemia, encephalopathy, and shock. Pediatr Crit Care Med. (2023) 24(10):795–806. 10.1097/PCC.0000000000003292 37272946 PMC10540758

[60] Atreya MR Bennett TD Geva A Faustino EVS Rogerson CM Lutfi R Novel data-driven sepsis phenotypes in children study and the genomics of pediatric septic shock investigators. Biomarker assessment of a high-risk, data-driven pediatric sepsis phenotype characterized by persistent hypoxemia, encephalopathy, and shock. Pediatr Crit Care Med. (2024) 25(6):512–7. 10.1097/PCC.0000000000003499 38465952 PMC11153020

[61] Zampieri FG Bagshaw SM Semler MW. Fluid therapy for critically ill adults with sepsis: a review. JAMA. (2023) 329(22):1967–80. 10.1001/jama.2023.7560 37314271

[62] Zarbock A Kellum JA Schmidt C Van Aken H Wempe C Pavenstädt H Effect of early vs delayed initiation of renal replacement therapy on mortality in critically ill patients with acute kidney injury: the ELAIN randomized clinical trial. JAMA. (2016) 315(20):2190–9. 10.1001/jama.2016.5828 27209269

[63] González R Urbano J López-Herce J. Resuscitating the macro- vs. microcirculation in septic shock. Curr Opin Pediatr. (2024) 36(3):274–81. 10.1097/MOP.0000000000001345 38446225

[64] Hariri G Joffre J Deryckere S Bige N Dumas G Baudel JL Albumin infusion improves endothelial function in septic shock patients: a pilot study. Intensive Care Med. (2018) 44(5):669–71. 10.1186/2110-5820-2-S1-S15 29392345

